# Iatrogenic central retinal artery occlusion following retrobulbar anesthesia with adrenaline for vitreoretinal surgery: a case report

**DOI:** 10.1186/s13256-022-03518-0

**Published:** 2022-08-09

**Authors:** Filippo Confalonieri, Gunn Elin Ladstein, Ingar Stene-Johansen, Goran Petrovski

**Affiliations:** 1 Department of Ophthalmology, Oslo University Hospital, Kirkeveien 166, 0450 Oslo, Norway; 2 Center for Eye Research, Department of Ophthalmology, Institute for Clinical Medicine, University of Oslo, Kirkeveien 166, 0450 Oslo, Norway; 3 Department of Biomedical Sciences, Humanitas Huniversity, Via Rita Levi Montalcini 4, 20090 Pieve Emanuele, Milan Italy; 4 Department of Ophthalmology, University of Split School of Medicine and University Hospital Centre, Split, Croatia

**Keywords:** Ophthalmology, Retina, Central retinal artery occlusion, Anesthesia, Adrenaline, Ocular surgery

## Abstract

**Background:**

We describe a patient presenting with central retinal artery occlusion (CRAO) of the right eye after retrobulbar anesthesia with adrenaline for macular pucker surgery.

**Case presentation:**

The patient, a 67-year-old Caucasian man, developed a CRAO postoperatively by the next-day control likely due to the retrobulbar injection of a combination of Xylocaine and Bupivacaine with adrenaline as anesthetic.

**Conclusions:**

The addition of adrenaline to the standard anesthetic solution could be a risk factor for serious complications, such as CRAO.

## Background

Central retinal artery occlusion (CRAO) has been reported as a rare complication associated to intraocular surgery in different types of ophthalmic surgery [1–4]. CRAO associated to anesthesia administration has been reported after sub-tenon [5, 6], peribulbar [7–11] and especially retrobulbar injection [2, 12–16]. Visual recovery is consistently reported to be poor in these patients. Epinephrine (adrenaline) is usually injected either with lidocaine or its derivatives to prolong the effects of a local anesthetic. Adrenaline is generally recognized as also havinga vasoconstrictive effect that decreases bleeding and counteracts the vasodilator effects of lidocaine through its sympathectomy effect. Eye vessels appear to be no exception even when anesthesia is administered outside the orbit [17–22]. Here, we report a case of a 1-day postoperative unilateral CRAO after vitreoretinal surgery with anesthetic containing adrenaline delivered by retrobulbar injection.

## Case presentation

A 67-year-old Caucasian man with a history of non-pathological myopia underwent uneventful surgery for macular pucker with epiretinal membrane (ERM) peeling in his left eye. Three months later, he underwent the same surgical procedure using the same retrobulbar anesthesia in his right eye. Preoperative best corrected visual acuity (BCVA) was 0.5 (− 1.25 sphere − 0.75 cylinder at axis 50) in the right eye and 0.75 (− 3.25 sphere − 1.75 cylinder at axis 95) in the left eye. Both eyes were pseudophakic at the time of macular pucker diagnosis and underwent the same procedure 3 months apart. The patient underwent surgery under monitored anesthesia care with a retrobulbar block using a 25-gauge (G), 38-mm Atkinson needle containing 5 ml of a 1:1 mixture of 2% Xylocaine containing adrenaline (1:200,000) and Bupivacaine 5 mg/ml. Both eyes were operated by the same experienced surgeon. In both cases the retrobulbar anesthesia was administered by the same experienced ophthalmologist and the same drug combination was used.

Preoperative review of the patient’s medical history showed that the patient was under observation due to a myocardial infarction that he had about 5 years previously. He also was undere rheumatological observation for ankylosing spondylitis. His treatment at the time of surgery consisted of acetylsalicylic acid 75 mg once daily and atorvastatin 40 mg once daily. No other health problems were reported. The patient denied any allergies. The patient’s social history was negative for smoking, alcohol abuse, recreational drug use, and travel abroad. The patient was a doctor who had been worked in the hospital as a clinician for about 30 years. His mother suffered from migraines and died of a heart attack at the age of 70 years. At the age of 69 years, his maternal grandfather suffered a stroke. The patient did not know anything about his father’s side of the family, but there was no other family history of stroke or vascular illness.

Three 25G trocars were placed through a self-sealing sclerotomy construction. Central and peripheral pars plana vitrectomy (PPV) was performed. Preexisting posterior vitreous detachment (PVD) induction was verified. Brilliant Blue G containing dye (ILM-BLUE®; D.O.R.C., Zuidland, the Netherlands) aided visualization of the internal limiting membrane (ILM) and allowed for both ERM and ILM peeling up to the vascular arcades. Peripheral indentation allowed for retinal lesion verification. No breaks were found. BSS intraocular irrigating solution was left in the vitreous chamber. The sclerotomies were self-sealing and no sutures were needed. At the conclusion of the procedure, about 0.2 mg of subconjunctival gentamycin was administered. No gas bubble was instilled, there were no episodes of hypotension during the surgery, and postoperatively the patient did not sleep in the prone position.

**Fig. 1:**
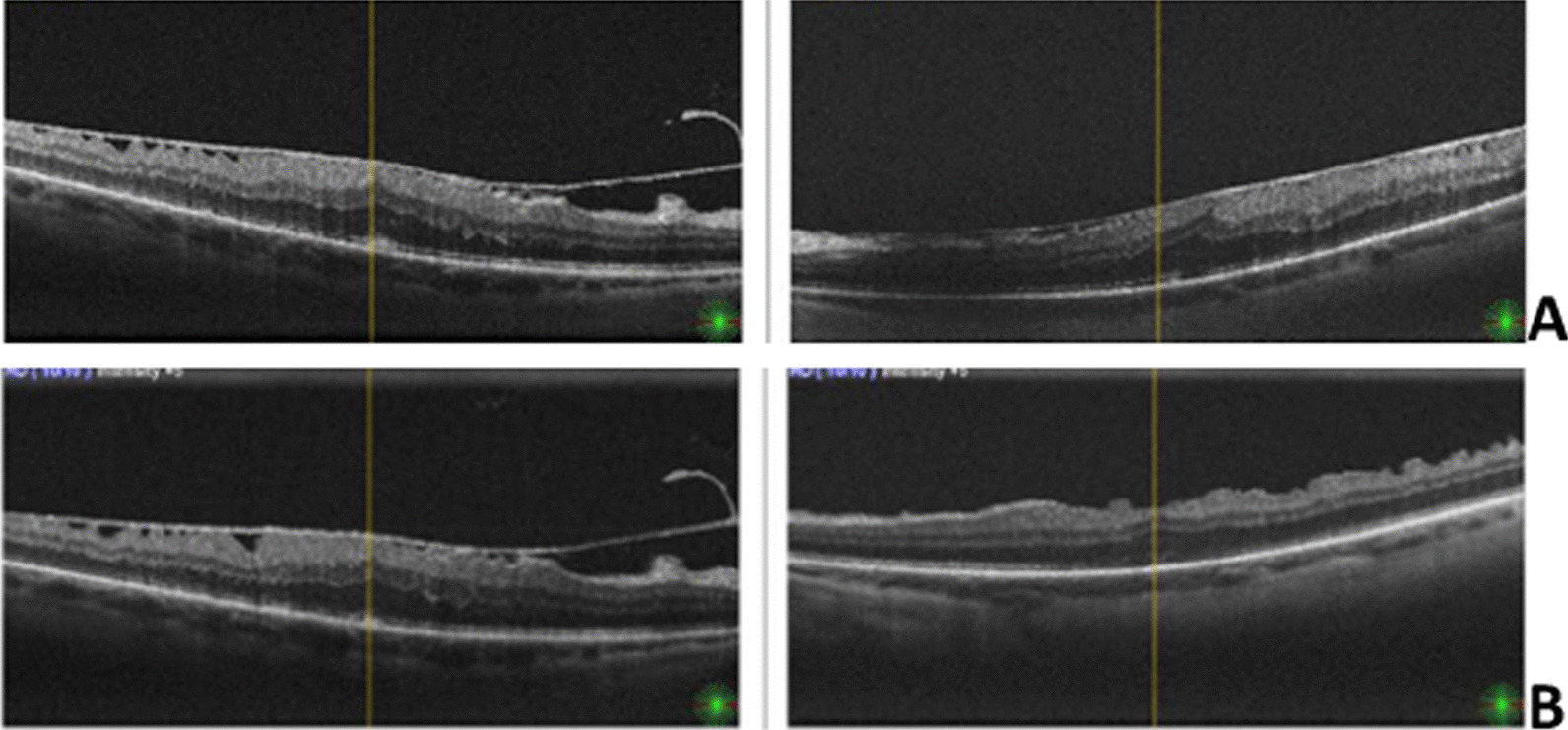
One day after uncomplicated left eye pars plana vitrectomy (PPV) + epiretinal membrane (ERM) peeling procedure. **a** Preoperative cross-sectional optical coherence tomography (OCT) scan of both eyes showed macular ERM. **b** Three months postoperative cross-sectional OCT scan of the left eye shows release of ERM-related anteroposterior traction

**Fig. 2:**
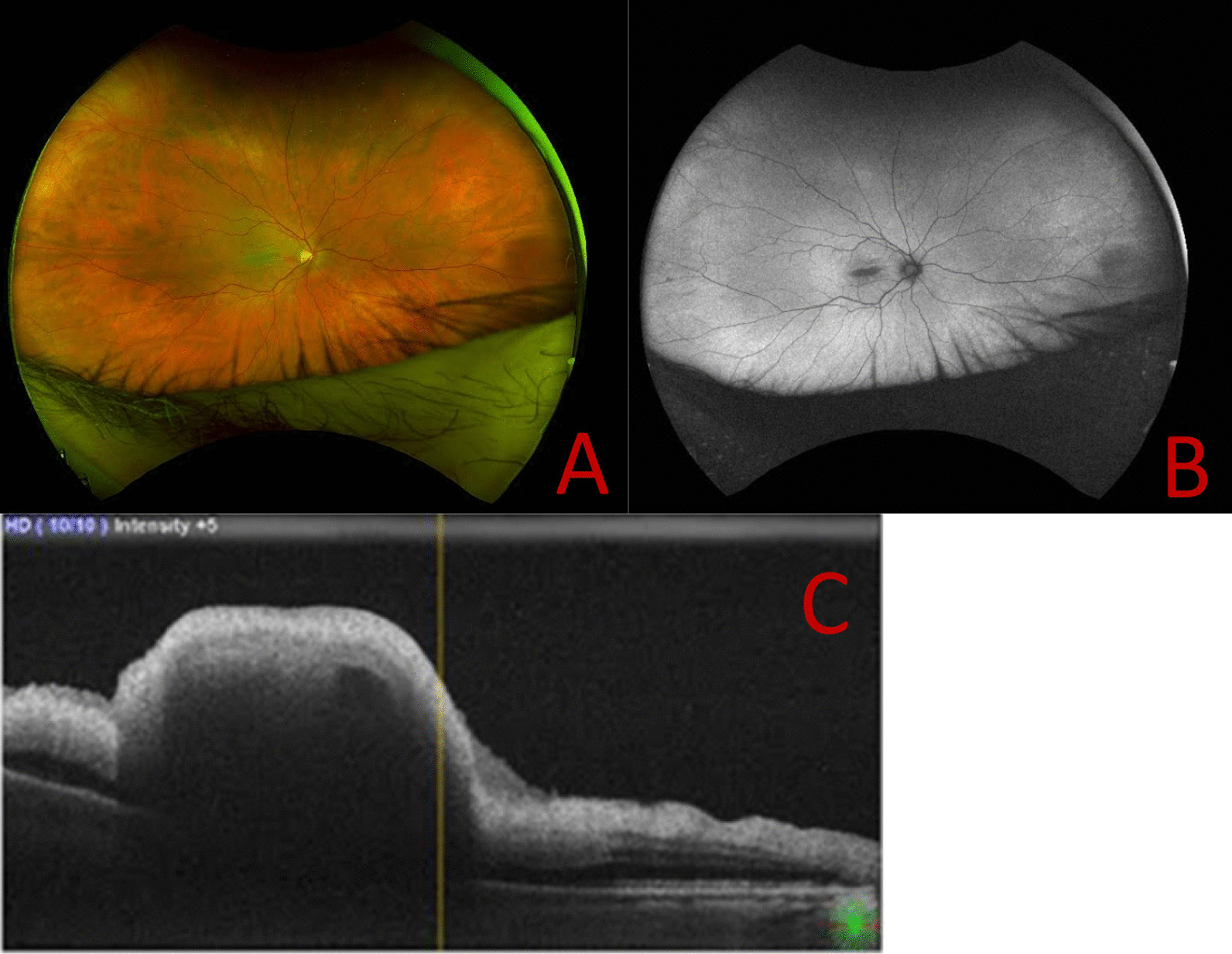
Right eye central retinal artery occlusion (CRAO) 1 day after uncomplicated PPV + ERM peeling procedure. **a** One-day postoperative wide-field fundus photograph of right eye showed CRAO. **b** One-day postoperative fundus autofluorescence. **c** One-day postoperative macular cross-sectional OCT shows foveal ischemia and edema

The left eye had a regular postoperative course (Fig. 1a, b). On postoperative day 1 the patient was seen by a junior ophthalmologist, and the visual acuity (VA) in the right eye was hand motion. Intraocular pressure (IOP) was 14 mmHg. There was a trace afferent pupillary defect by reverse in the right eye. The posterior segment examination showed retinal whitening in the macula and a cherry-red spot (Fig. 2a, b).

The retina was attached. There was no proptosis or orbital fullness. Optical coherence tomography (OCT) demonstrated inner retinal thickening and hyperreflectivity (Fig. 2c). Fluorescein angiography demonstrated delayed arterial and venous perfusion (not shown).

No further systemic evaluation was performed, the patient was observed and was not referred to a stroke center by the junior ophthalmologist. No blood tests were carried out. No blood pressure, pulse nor temperature was recorded. The patient was not treated with anterior chamber paracentesis, timolol-dorzolamide and brimonidine drops, or 500 mg oral acetazolamide at the first day postoperative control. The patient was alert, attentive, and oriented. Speech was clear and fluent. Cranial nerve assessment, reflexes, sensory perception, coordination, and gait were all normal. No signs of cerebrovascular event were reported.

**Fig. 3:**
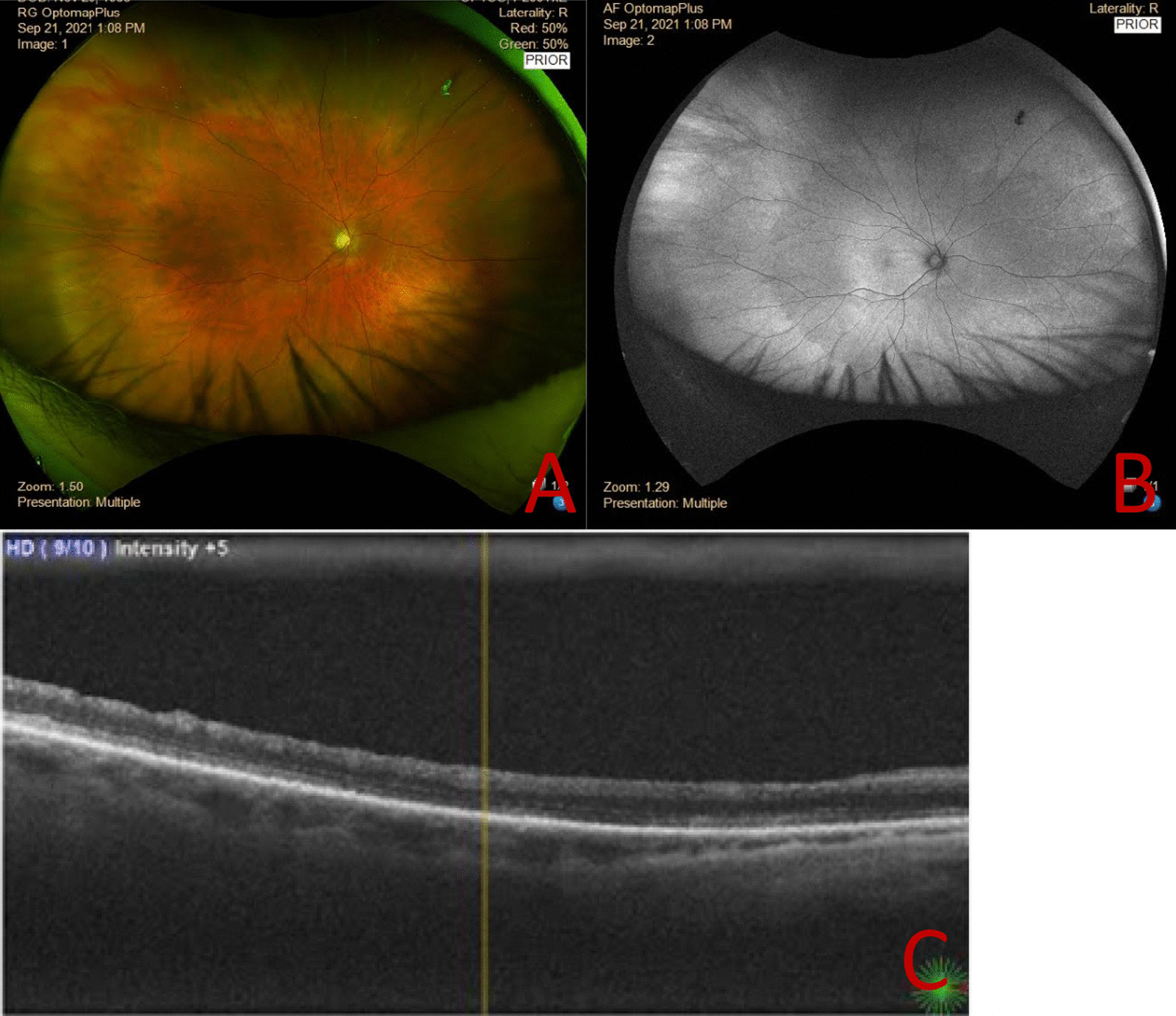
Right eye CRAO 3 months after PPV + ERM peeling procedure. **a** Three-month postoperative wide-field fundus photograph and of right eye showed reduction of retinal whitening and disappearance of cherry red spot. **b** Three-month postoperative fundus autofluorescence image. **c** Three-month postoperative macular cross-sectional OCT shows atrophic foveal region and difficulty in central fixation

At 3 months after surgery, the patient felt his central scotoma had improved, and the BCVA had remained stable at 0.05 (− 0.75 to 1.0 axis 60). The anatomical signs of acute ischemia had resolved, and the macular region resulted in atrophic changes with disappearance of the physiological foveal depression (Fig. 3).

**Fig. 4:**
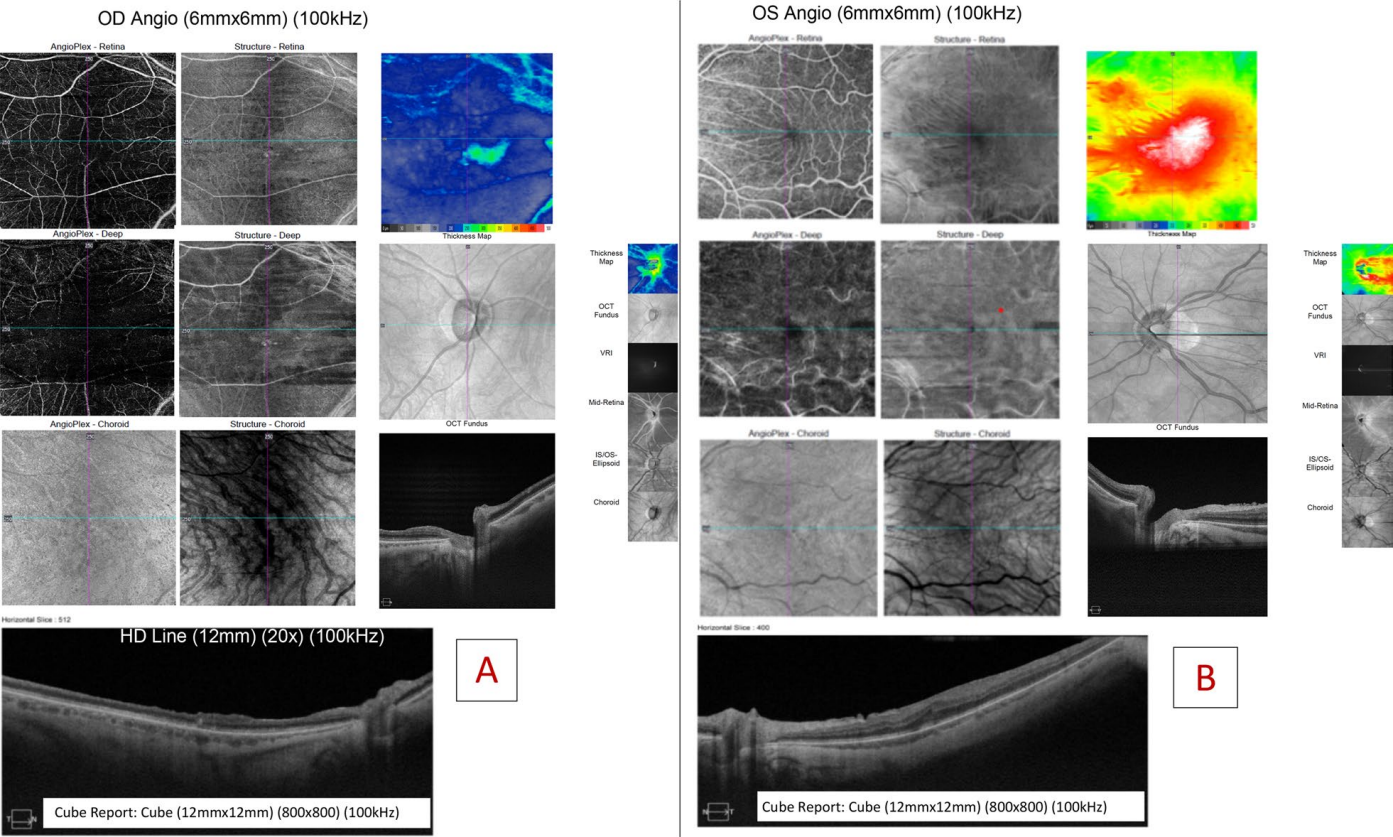
Right eye CRAO 4 months after uncomplicated PPV + ERM peeling procedure. **a** 4 month-postoperative right-eye whole retinal, deep, and choroidal OCT angiogram show retinal ischemia predominantly in the deep plexus. HD structural line OCT scanning the fovea shows atrophy. **b** The left eye is shown for comparison. The left eye underwent the same procedure without any complication

At 4 months after surgery, OCT angiography documented a right eye capillary dropout predominantly in the deep capillary plexus (Fig. 4)

## Discussion and conclusions

We report a patient with CRAO that occurred in the postoperative period after vitreoretinal surgery with PPV + ERM peeling. Both eyes underwent the same procedure 3 months apart by the same experienced surgeon, but only the second eye showed CRAO.

Many authors have previously described the occurrence of CRAO after retrobulbar anesthesia, as summarized in Table 1.

**Table 1:** Summary of previous reports of central retinal artery occlusion after retrobulbar anesthesia

Study	Age (years), gender	Cardiovascular risk factors	Diagnosis	Procedure	Therapy	Time to treatment or examination (days)	Preoperative Visual Acuity	Postoperative Early Visual Acuity	Postoperative Final Visual Acuity	Follow-up (days)
Klein *et al.* [14]	19, F 43, F 33, F 59, M	DM Sickle-cell hemoglobinopathy Sickle-cell hemoglobinopathy Carotid insufficiency	PDR SCR SCR OIS with secondary glaucoma	Photocoagulation Photocoagulation Photocoagulation Photocoagulation	Ocular massage Ocular massage None AC paracentesis	Immediately Immediately Immediately	20/50 NA 20/40 20/50	FC HM FC NA	20/30 20/20 20/50	7 3 1
Sullivan *et al.* [36]	60, M 81, F 67, F	Acetylsalicylic acid daily Acetylsalicylic acid daily NA	Cataract Cataract AACG	Phaco + IOL Cataract extraction with AC IOL implantation Surgical iridectomy	Kronlein lateral orbitotomy with nerve sheath decompression, carbogen inhalation, lowering IOP Mx None None	6 2 4	20/60 NA 20/32	20/60 NLP 20/200	6/60 NLP	6 0 7
Cowley *et al.* [35]	30, F	DM	PDR	Photocoagulation	Ocular massage, AC paracentesis, sublingual nitroglycerin, inhalation of carbon dioxide	Immediate	20/80	NA	NA	NA
Mieler *et al.* [15]	34, F	None	Cataract	Phaco+IOL	None	1	NA	LP	HM	150
Roth *et al.* [34]	38, M	Sickle-cell hemoglobinopathy	Proliferative SCR	Photocoagulation	None	Immediately	NA	HM	HM	365
Giuffre *et al.* [13]	61, M	HTN	Cataract	Extracapsular cataract extraction	None	3	NA	LP	NLP	14
Torres *et al.* [33]	74, F 66, F	None HTN	Cataract Cataract	Extracapsular cataract extraction with IOL implantation Extracapsular cataract extraction with IOL implantation	None None	1 1	1	NA NA	LP LP	CRVO CRVO
Mameletzi *et al.* [32]	78, F	None	Cataract	Phaco + IOL	Lowering IOP Mx, anticalcic therapy, methylprednisolone	1	NA	LP	HM	30
Tappeiner *et al.* [31]	58, M 79, M 83, F	HTN HTN, aorta aneurysm None	Macular pucker Macular hole Vitreous hemorrhage	Vitrectomy Vitrectomy Vitrectomy	Lowering IOP Mx Lowering IOP Mx Lowering IOP Mx	2-14 2-14 2-14	NA NA NA	NA NA NA	20/320 LP 20/200	365 365 365
Jung *et al.* [2]	72, M 72, F 53, F 72, M 66, F	HTN, cerebral infarction HTN, DM, ICA stenosis, MI HTN, DM, ESRD, cerebral infarction HTN HTN	Cataract VH VH Macular pucker Cataract	Phaco + iol PPV PPV PPV Phaco + IOL	Intraarterial thrombolysis Intraarterial thrombolysis Anteriorchamber paracentesis, lowering IOP Mx None intraarterial thrombolysis	1 1 7 1 1	NLP NLP HM HM HM	NA	NLP LP NLP FC FC	1367 4 1577 1807 942
Vasavada *et al.* [4]	65, F 46, F	DM, HTN None	Cataract Cataract	MSICS Phaco + IOL	Ocular massage, AC paracentesis, IOP lowering drops None	1 30	NA NA	NA NA	CF CF	NANA
Fischer *et al.* [30]	72, F 63, M 69, M	None DM None	MH with several peripheral retinal degenerations Cataract RRD	Encircling band, PPV, C2F6 (10%) Phaco + IOL PPV, encircling band, C2F6 (14%)	None None None	19 69 21	0.4 0.5 0.1	NA NA NA	HM 1.0 HM	NA NA NA
Russell *et al.* [1]	28, M 70, F	Familiarity for MI HTN	RRD Vitrous opacities	PPV	AC tap, timolol-dorzo., brimon., hyper-ventilation None	1 1	20/15 20/25	LP 3/200		300 180
Confalonieri *et al.* [29]	67, M	History of MI	Macular pucker	PPV + peeling ERM	None	1	0.5	0.05		120

*AC* anterior chamber, *CF* counting finger, *DM* diabetes mellitus, *ERM* epiretinal membrane, *ERSD* end-stage renal disease, *F* female, *FC* finger counting, *HM* hand motion, *HTN* arterial hypertension, *ICA* internal carotid artery, *IOL* intraocular lens, *IOP* intraocular pressure, *LP* light perception, *M* male, *MH* macular hole, *MI* myocardial infarction, *NLP* no light perception, *OIS* ocular ischemic syndrome, *PPV* pars plana vitrectomy, *RRD* regmatogenous retinal detachment, *SCR* sickle cell retinopathy

CRAO is a known, but very rare complication of ocular surgery that can occur after retrobulbar, peribulbar, or sub-Tenon’s anesthesia [1–14]. It is also a known, but very rare complication of adrenaline injection as an adjuvant in anesthesia administration in other parts of the body, especially in ear, nose and throat, oral, and plastic surgery [14–20]. To our knowledge, this is the first report to associate retrobulbar anesthesia injection combined with adrenaline to CRAO.

We suspect that multiple factors related to the adrenaline injection might have contributed to the development of this case. Since CRAO can happen after retrobulbar anesthetic injection even in absence of adrenaline, however, this might just be one of those rare cases of increased intraorbital pressure in a patient affected by vasculopathy resulting in ischemia.

Since adrenaline can cause CRAO following trigeminal nerve block during oral procedures or local anesthesia of the nasal mucosa during nasal surgery [14–20], the proposed mechanism is arterial occlusion resulting from either direct or indirect mechanical trauma with subsequent vasospastic events or intraarterially injected adrenaline with retrograde migration [20, 23–27]. The Atkinson needle has a blunt tip and would be expected to cause minimal trauma to the surrounding tissue.

Adrenaline acts peripherally on α-adrenergic receptors [28], resulting in the constriction of blood vessels. Thus, in our case, retrograde arterial migration of the injected adrenaline into the ophthalmic arterial system might have blocked the ophthalmic artery immediately after injection. Through vasodilation over time, subsequent anterior movement of adrenaline to more distal vessels may have led to vasoconstriction and subsequent vasospasm.

We exclude the hypothesis of allergic reaction to adrenaline, even though sensitization could have happened after the first vitreoretinal operation, because of lack of systemic symptoms.

Adrenaline can lead to CRAO following retrobulbar injection of intraconal administered local anesthetics. Hence, physicians should carefully administer local anesthesia with adrenaline in the intraconal space while considering the possibility that such a complication may occur, or possibly exclude anesthetics containing adrenaline during retrobulbar anesthesia.

## Data Availability

Data and material can be found at the Oslo University Hospital, Ophthalmology Department.

## Abbreviations

BCVA: Best corrected visual acuity
CRAO: Central retinal artery occlusion
ERM: Epiretinal membrane
G: Gauge
ILM: Internal limiting membrane
IOP: Intraocular pressure
PPV: Pars plana vitrectomy
PVD: Posterior vitreous detachment
VA: Visual acuity
